# Removal of Cr(vi) from wastewater by a two-step method of oxalic acid reduction-modified fly ash adsorption

**DOI:** 10.1039/c9ra05980f

**Published:** 2019-10-22

**Authors:** Xiaoling Jiang, Wenqiang Fan, Chunqing Li, Yong Wang, Junbin Bai, Hongjian Yang, Xiaoli Liu

**Affiliations:** Hebei Provincial Key Laboratory of Green Chemical Technology and High Efficient Energy Saving, Tianjin Key Laboratory of Chemical Process Safety, School of Chemical Engineering and Technology, Hebei University of Technology Tianjin 300130 China 1991033@hebut.edu.cn +86-13132143286; Chengan Thermal Power Co., Ltd Tianjin 300204 China 513046743@qq.com +86-18722287350

## Abstract

Excessive Cr(vi) emissions have been and continue to be a major contributor to heavy-metal pollution; recently, the development of a low-cost, safe and efficient method for the removal of Cr has attracted significant attention. In the present study, a two-step method involving oxalic acid reduction and modified fly ash adsorption was developed. The experimental results showed that this methodology exhibited high Cr(vi) removal efficiency under the following conditions: 1.5 g L^−1^ of oxalic acid, modification of fly ash (FA) by 20 wt% KOH, a contact time of 2 h and a mass of 0.3 g of modified fly ash (MFA) at room temperature (15–25 °C). The influencing factors of the adsorbent were discussed by characterized for their elemental composition, functional groups, surface area and surface morphology. According to the characteristic parameters and *q*_e_, the isothermal adsorption process could be well-described by the Langmuir model. The adsorption process resembles more closely to the pseudo-second-order kinetic model. In conclusion, this two-step method of oxalic acid reduction-modified fly ash adsorption is promising for Cr(vi) removal.

## Abstract 2

Removal of Cr(vi) from wastewater using a two-step method.

## Graphical abstract

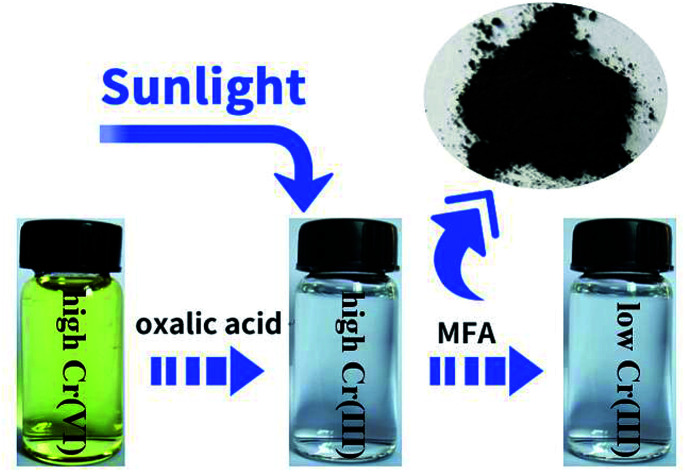

## Introduction

Heavy-metal pollution in groundwater and surface water continues to be a serious environmental challenge. Unfortunately, the presence of heavy metals, even at small concentrations, in usable water is detrimental to human health; moreover, chromium compounds that are widely used in several industrial processes, such as printing, electroplating, leather tanning, polishing and pigment industries, lead to the emission of Cr(vi) and thus contribute to heavy-metal pollution. More specifically, the wastewater produced by these industries typically contains Cr(vi) at high concentrations. Cr exists primarily in two states: Cr(iii) and Cr(vi). Although Cr(iii) has low toxicity and is essential in trace quantities to human beings, Cr(vi) is very toxic to human beings.^1^ In fact, Cr(vi) has been categorized as a group I human carcinogen by the International Agency for Research on Cancer. Thus, any reaction that can reduce Cr(vi) to Cr(iii) is strongly sought after.

Nowadays, there are two principal methods to reduce the Cr(vi) content in water. First, the addition of reducing agents to reduce the highly toxic Cr(vi) to its low toxic counterpart, Cr(iii).^2–4^ However, this method fails to remove Cr from the system, and thus, Cr(iii) can eventually oxidize back to Cr(vi) in the presence of oxides and dissolved oxygen. Moreover, the cost to remove the Cr precipitate is currently very high.^5^ The other method involves the use of biomass adsorbents, such as peanut shells, banana peels,^6,7^ and mineral adsorbents, such as bentonite, montmorillonite, and medical stone, to absorb Cr(vi) and reduce its concentration in the wastewater. This method is facile to implement and possesses high adsorption efficiency. However, it is inadequate for reducing the toxicity of Cr(vi).

Oxalic acid is an economically efficient, abundant and naturally existing organic acid.^8^ Oxalic acid, as a reducing agent, can readily convert Cr(vi) to Cr(iii). However, the rate of reduction of Cr(vi) is very slow, and the half-life of these reactions ranges from months to several years.^9^ Consequently, significant research efforts have been directed towards improving the reaction rate of oxalic acid in the reduction of Cr(vi).^10^ For example, under specific conditions, MnO_2_ can accelerate the reduction of Cr(vi) in the presence of oxalic acid and also produce a stable complex.^11^

FA, the principle solid waste discharged from coal-fired power plants, is one of the largest contributors to China's growing carbon footprint. Moreover, the improper disposal of FA leads to damaged lands and poisoned environments. However, recent studies have stated that MFA can effectively remove metal ions from wastewater; more specifically, the removal efficiencies of Cr(vi), Hg(ii), Zn(ii), Ni, Pb, Fe, Mn, Al and COD have been greatly enhanced after the modification of fly ash. Therefore, rather than simply disposing FA, it can be modified as the MFA exhibits potential applications.^12–16^ Excellent adsorption coupled with its inexpensive abundance has re-enabled its potential in the recovery of heavy-metal-contaminated water. Therefore, by combining oxalic acid and FA, Cr(vi) can be removed from wastewater and its environmental toxicity can be reduced simultaneously.

In this study, we investigated a two-step method to eliminate Cr(vi) from wastewater; in detail, the first step included the reduction of Cr(vi) to Cr(iii) in the presence of oxalic acid, followed by the adsorption of Cr(iii) onto MFA. At first, the optimal concentration of oxalic acid was measured. Then, the removal efficiencies of Cr from the wastewater with different modification ratios of KOH and dosages of MFA were also optimized. Moreover, the physicochemical properties of FA before and after the modification were compared. Lastly, the mechanism of adsorption of Cr on MFA was explored to provide a rational basis for the application of oxalic acid/MFA in the treatment of heavy-metal-containing water.

## Materials and methods

### Materials

FA was obtained from the Yangliuqing Thermal Power Plant of Tianjin. Cr(vi) was prepared at 100 mg L^−1^. Potassium dichromate (K_2_Cr_2_O_7_), hydrochloric acid (HCl), sulfuric acid (H_2_SO_4_), nitric acid (HNO_3_), sodium hydroxide (NaOH), and oxalic acid (H_2_C_2_O_4_) were of analytical grade. Lastly, the water used in this study was deionized water unless stated otherwise.

### Treatment of contaminated water

The initial concentration of Cr(vi) was 100 mg L^−1^; wastewater and an oxalic acid solution in the ratio of 1 : 1 were mixed evenly, and then, the mixture was placed under sunlight. The concentrations of oxalic acid added to the solution mixture were 0.5, 1, 1.5, 2, 2.5, 3, 3.5 and 4 g L^−1^; the concentration of Cr(vi) was measured using the 1,5-diphenylcarbazide spectrophotometric method, whereas the concentration of Cr was measured by atomic absorption spectroscopy.

### Preparation of MFA

FA was rinsed to remove excess dirt and then dried in a vacuum oven for 12 h at 105 °C. After this, FA in a certain quantity and the KOH solution (100 mL) were mixed thoroughly.

**Fig. 1:**
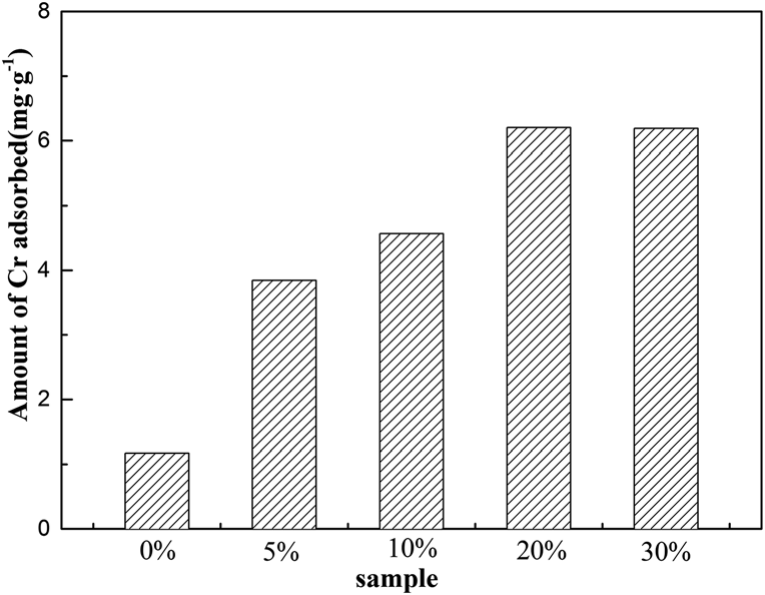
The adsorption capacity of Cr on MFA prepared using different concentrations of KOH.

Batch experiments using KOH at various concentrations (5 wt%, 10 wt%, 20 wt%, and 30 wt%) were conducted under different conditions to ensure that FA was modified; moreover, the best concentration of KOH for the preparation of MFA was found to be 20 wt%. Therefore, MFA used in the following experiments was modified with 20 wt% KOH (Fig. 1).

### Characterization and analysis

For crystal-phase composition analysis, the XRD patterns of FA and MFA were obtained *via* the D8 focus powder diffractometer (Bruker, Germany) using Cu Kα radiation with the tube voltage of 40 kV and tube current of 40 mA. Data was obtained at an increment of 0.019° and the scanning speed of 5 degrees s^−1^. The quantitative phase analysis was performed by the Rietveld method using the Topas5.0 software package. Moreover, the micro-morphology of the fractured surface of MFA was investigated *via* the Nova Nano SEM450 field-emission electron microscope (FESEM) obtained from FEI using an acceleration voltage of 1.00 kV. An FTIR spectrum was obtained by the Tensor27 Fourier-transform infrared spectrometer (Bruker, Germany) using thin films prepared on KBr. Lastly, the element and valence of FA and MFA were analyzed by X-ray photoelectron spectroscopy (Thermofisher, America) using Al Kα radiation with a full-spectrum pass energy of 100.0 eV, step size of 1.00 eV, narrow-spectrum pass energy of 30.0 eV, step length of 0.05 eV, and a binding energy that was corrected based on the binding energy of C 1s (binding energy = 284.8 eV).

### Adsorption experiments

The batch experiments were carried out by mixing MFA in various amounts with 50 mL of KOH solution at predetermined concentrations at room temperature (15–25 °C) using a reciprocal shaker bath at 90 rpm; then, the solution was filtered, leaving the residual metal in the supernatant, which was analyzed *via* atomic absorption spectroscopy. The removal efficiency *η* and the adsorption capacity were computed using the eqn (1) and (2), respectively:1
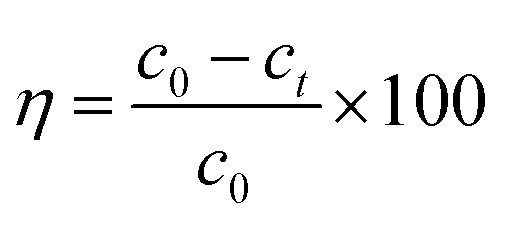
2
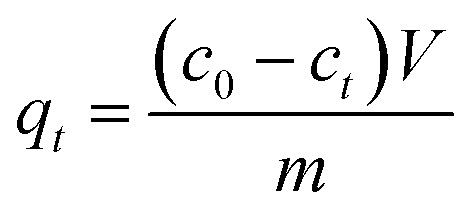
where *q*_e_ (mg g^−1^) is the equilibrium adsorption capacity of MFA, *c*_0_ (mg L^−1^) is the initial equilibrium concentration of Cr, *c*_t_ (mg L^−1^) is the equilibrium concentration of Cr, *V* (L) is the volume of the aqueous solution, and *m* (g) is the adsorbent mass.

### Adsorption kinetics

To probe the MFA adsorption kinetics, 0.2 g of MFA and 50 mL of the treated solution were added to a 250 mL Erlenmeyer flask at room temperature (15–25 °C). For comparison, a blank experiment was carried out using FA.

Moreover, three kinetic models (pseudo-first-order, pseudo-second-order, and intra-particle diffusion models) were used to fit the experimental data:

Pseudo-first-order model:

3 ln(*q*_e_ − *q*_t_) = ln *q*_e_ − *k*_1_*t*

Pseudo-second-order model:4
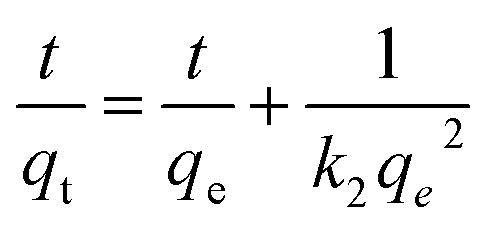


Intra-particle diffusion model:

5 *q*_t_ = *k*_p_*t*^0.5^ + *C*

where *k*_1_ (h^−1^) is the first-order rate constant, *k*_2_ (g mg^−1^ h^−1^) is the second-order rate constant, *k*_p_ (mg g^−1^ h^−0.5^) is the intra-particle constant, *t* (h) is the adsorption time, and *q*_e_ and *q*_t_ (mg g^−1^) are the adsorption capacities of the adsorbent at equilibrium and the time *t* (h), respectively.

### Studies of the adsorption isotherm models

A series of experiments were setup with various initial Cr concentrations (from 5 mg L^−1^ to 150 mg L^−1^) for 24 h at room temperature (15–25 °C) using the MFA dosage of 0.2 g, and FA instead of MFA was used in the blank experiment.

To determine the maximum capacity of MFA, the equilibrium isotherm data were modelled by the Langmuir and Freundlich isotherms. These equations can be written as follows:

The Langmuir isotherm-linearization is as follows:6
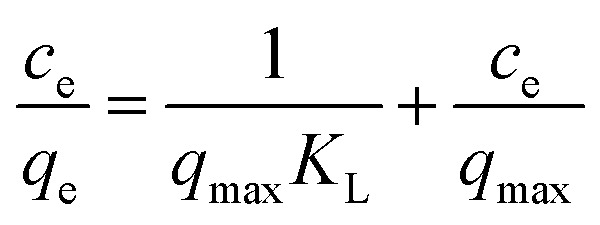


The Freundlich isotherm-linearization is as follows:7
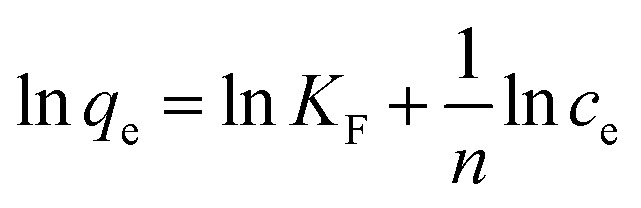
where *q*_e_ (mg g^−1^) is the Cr adsorption capacity of the adsorbate (mg g^−1^) at equilibrium and *q*_max_ (mg g^−1^) is the saturated adsorption capacity.

## Results and discussion

### Effect of oxalic acid concentration

**Fig. 2:**
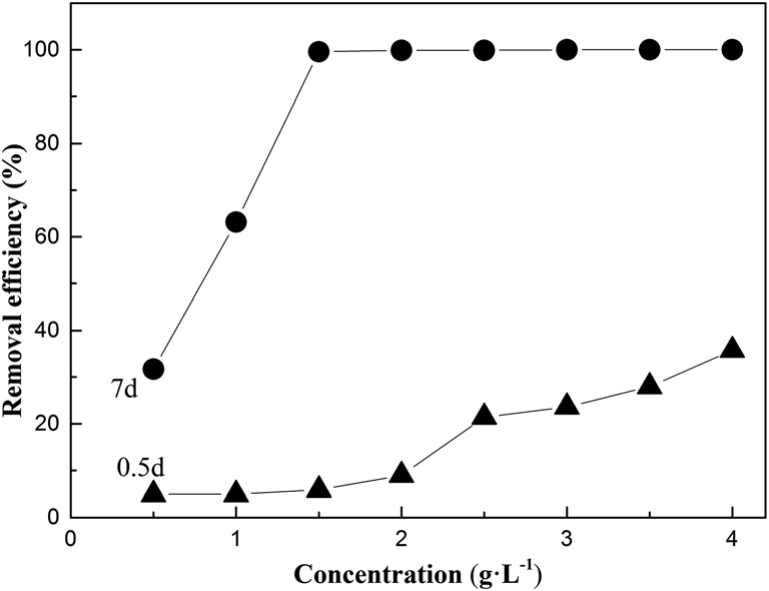
The effect of oxalic acid concentration on the removal efficiency after 0.5 d and 7 d (under sunlight; the mixed concentration of Cr(vi) = 50 mg L^−1^ and *T* = 15–25 °C).

Fig. 2 shows the effect of the stability and concentration of oxalic acid on the removal efficiency of Cr(vi) under sunlight. The results demonstrated that the optimum concentration of oxalic acid was 1.5 g L^−1^, and oxalic acid reacted with Cr(vi) to form a monovalent oxalate complex (C_2_CrO_7_^2−^) by esterification. It has been reported that the transfer of oxalate electrons to the chromate moiety occurs by charge excitation transfer to achieve the reduction of Cr(vi) to Cr(iii) under sunlight, and Cr(iii) can complex with the oxalic acid component to form Cr(iii)-oxalate complex products.^17^ The concentration of Cr(vi) was measured using the 1,5-diphenylcarbazide spectrophotometric method. After 7 days, the presence of Cr(vi) was reduced greatly, indicating that oxalic acid reduced Cr(vi) to Cr(iii) and the Cr(iii)–oxalate complex was very stable.^11,18,19^

8 2HCrO_4_^−^ + 3HC_2_O_4_^−^ + 11H^+^ → 2Cr^3+^ + 6CO_2_ + 8H_2_O

9 Cr^3+^ + 3HC_2_O_4_^−^ → Cr(HC_2_O_4_)_3_

### Characterization of the adsorbent

**Fig. 3:**
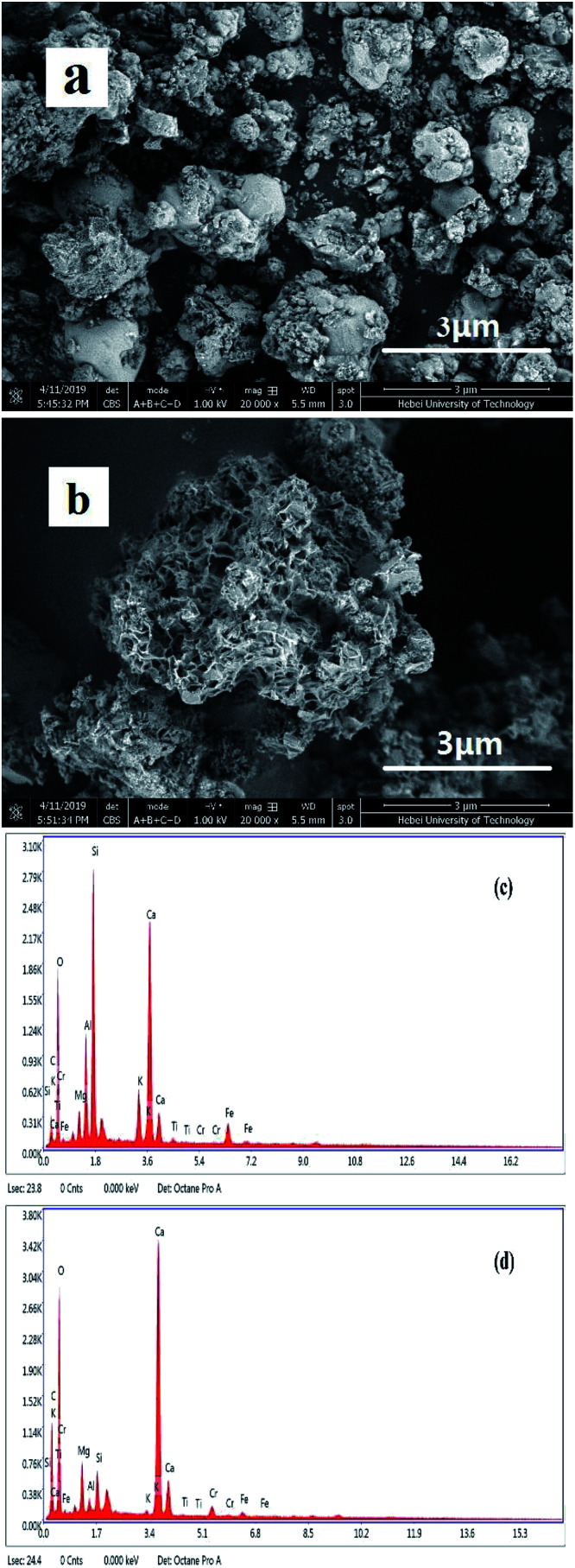
SEM images of (a) FA and (b) MFA and EDS spectrum of MFA (c) before and (d) after Cr adsorption.

Fig. 3(a) and (b) show the surface morphologies of FA and MFA and the EDS spectrum of MFA before and after the adsorption of Cr, obtained *via* SEM and EDS, respectively. Fig. 3(a) reveals that FA contains many glassy particles, and the surface of these particles contains tiny active channels. By comparing Fig. 3(a) and (b), it was found that the surface of MFA was rougher than that of FA, and the porous structure was more apparent; since potassium hydroxide destroyed the dense, vitreous texture of the FA surface, the activity of FA was enhanced due to the presence of more adsorption active centers for the adsorption process that increased the adsorption performance of FA.

The EDS spectrum (Fig. 3(c)) of MFA before the adsorption process confirmed the presence of C, O, Mg, Al, Si, K, Ca, Fe, and minute amounts of Cr (0.01%). In contrast, larger amounts of Cr (1.08%) were detected (Fig. 3(d)) after adsorption. The results confirmed that Cr readily adsorbed on the surface of MFA.

**Fig. 4:**
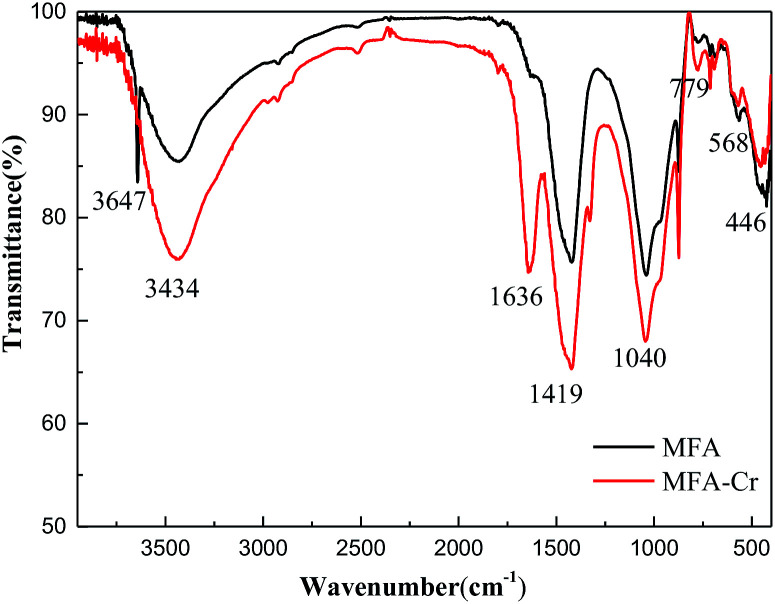
FTIR spectra of MFA before and after Cr adsorption.

The FTIR spectra of MFA before and after Cr adsorption are shown in Fig. 4. There are a large number of functional groups on the surface of FA and MFA, which play a crucial role in the process of adsorption of heavy metal ions.^20^ The broadband at 3434 cm^−1^ is attributed to the stretching vibrations of O–H.^21^ The peaks at 1636 cm^−1^ and 1419 cm^−1^ correspond to the C=C stretching bands in the aromatic ring.^22^ The strong peak at 1040 cm^−1^ is the result of the asymmetric stretching vibrations of Si–O–Si.^23^ The peak at 779 cm^−1^ is related to the Si–O–Si bending modes.^23^ The peaks at 568 cm^−1^ and 446 cm^−1^ correspond to silicate minerals and the network of Si–O–Si bond-bending vibrations.^24^ The intensity of the peaks in the FTIR spectra increased after Cr adsorption, and the positions of certain peaks shifted. Specifically, the sharp peak at 3647 cm^−1^ disappeared, and a new peak appeared at 1636 cm^−1^. Moreover, the intensity of the peaks at 1419, 1040, and 779 cm^−1^ significantly enhanced, whereas that of the peaks at 568 and 446 cm^−1^ reduced. These phenomena could be rationalized by speculating that Cr was bound possibly during the complexation process; in addition, the EDS measurements performed after adsorption confirmed the presence of Cr.

**Fig. 5:**
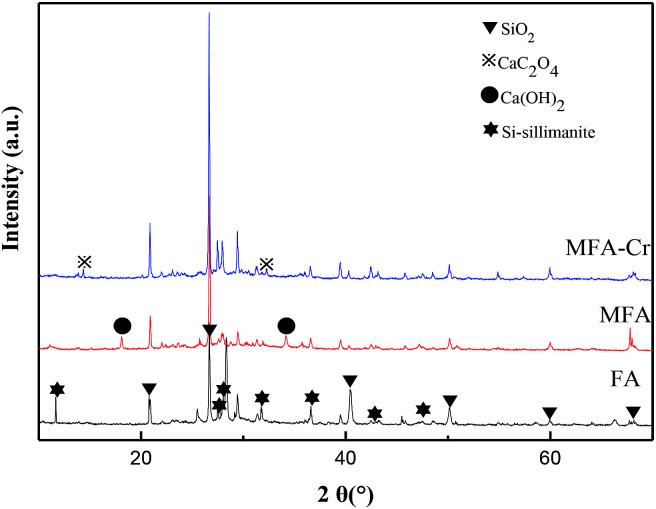
XRD pattern of MFA before and after Cr absorption.

The XRD spectra shown in Fig. 5 verify that FA is primarily composed of quartz, carbon and Si-sillimanite. Since potassium hydroxide is a strong base, it destroys the dense, vitreous surface of FA, forming new adsorption sites and enhancing the activity of FA. As a result, the specific surface area of MFA was estimated to be 10.38 m^2^ g^−1^, which was significantly large as compared to that of FA (6.43 m^2^ g^−1^). Larger surface areas imply the exposure of more active sites to the active material, which is more conducive to the formation of surface complexes; the charges at the active sites on the surface of MFA (eqn (10) and (11)), which allow Cr^3+^ to be complexed on the surface (eqn (12) and (13)), are as follows:^25^

10

<svg xmlns="http://www.w3.org/2000/svg" version="1.0" width="23.636364pt" height="16.000000pt" viewBox="0 0 23.636364 16.000000" preserveAspectRatio="xMidYMid meet"><metadata>
Created by potrace 1.16, written by Peter Selinger 2001-2019
</metadata><g transform="translate(1.000000,15.000000) scale(0.015909,-0.015909)" fill="currentColor" stroke="none"><path d="M80 600 l0 -40 600 0 600 0 0 40 0 40 -600 0 -600 0 0 -40z M80 440 l0 -40 600 0 600 0 0 40 0 40 -600 0 -600 0 0 -40z M80 280 l0 -40 600 0 600 0 0 40 0 40 -600 0 -600 0 0 -40z"/></g></svg>

SiOH + OH^−^ → SiO^−^ + H_2_O

11 AlOH + OH^−^ → AlO^−^ + H_2_O

12 3(SiO^−^) + Cr^3+^ → (Si–O)_3_Cr

13 3(AlO^−^) + Cr^3+^ → (Al–O)_3_Cr

Previous studies have shown that high contents of calcium ash and unburnt carbon are very favorable for good adsorption properties.^26,27^ In this study, CaO and Ca(OH)_2_ in MFA formed a precipitate with Cr^3+^ and HC_2_O_4_^−^ to reduce the content of Cr in the wastewater. The XRD spectra of MFA confirmed the formation of CaC_2_O_4_ on the surface of MFA after the adsorption process, and as a result, the amount of Cr in the wastewater was reduced greatly. Thus, HC_2_O_4_^−^ was removed by precipitation–adsorption and Cr was bound during the complexation process.

**Fig. 6:**
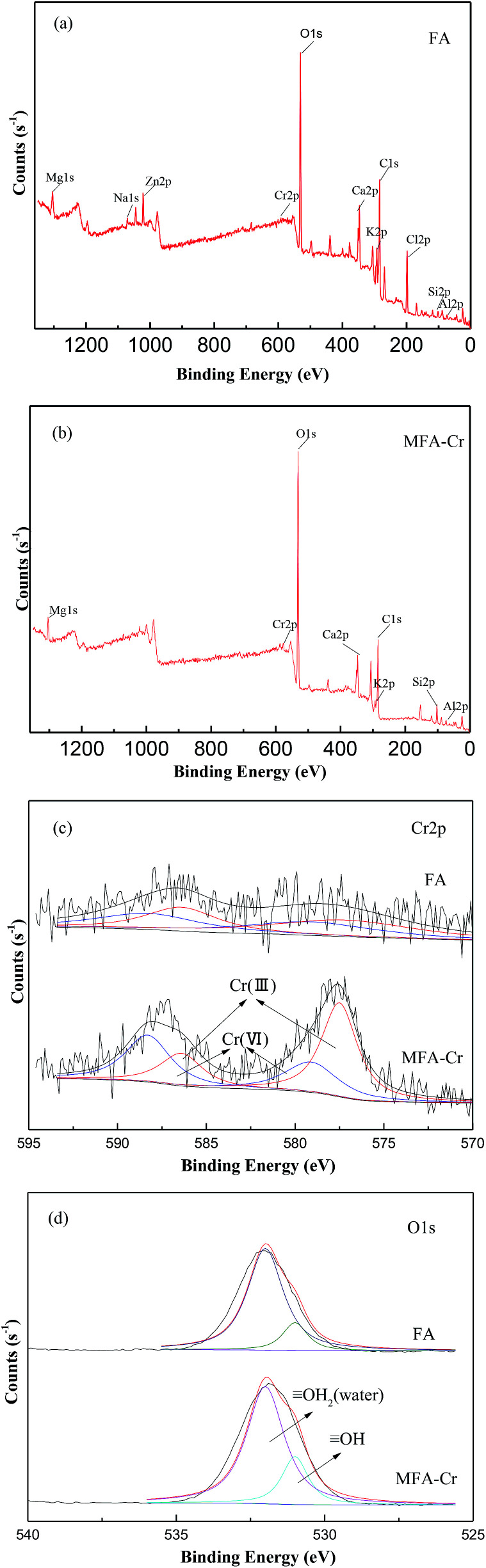
XPS spectra of MFA (a) before and (b) after Cr absorption and the XPS survey spectra of (c) Cr 2p and (d) O 1s.

XPS analysis was utilized to aid the elucidation of the absorption mechanisms of Cr on the surface of MFA. Fig. 6(a) and (b) present the entire region scan of MFA before and after being exposed to the wastewater having 50 mg L^−1^ of Cr; in particular, the photoelectron peaks at 577.2 eV and 586.4 eV are attributed to Cr 2p of Cr(iii) and the peaks at 588.3 eV and 579.1 eV are attributed to Cr 2p of Cr(vi). Fig. 6(c) displays the XPS survey spectrum for Cr 2p. It was found that the peak area ratio of Cr(iii) increased after adsorption, which proved that the Cr(iii) compounds were formed on the surface, and Cr(OH)_3_ exhibited a peak centred at 577.3 eV.^28,29^Fig. 6(d) exhibits an XPS survey spectrum of O 1s. The peaks can be fitted to the peaks at 530 eV, 531 eV, and 532 eV, which correspond to ≡O^−^, ≡OH, and water (≡OH_2_).^30^ Moreover, the peak area percentage of ≡OH increased after adsorption; this suggested that the chromium compounds that were formed on the surface were most likely Cr(OH)_3_. Lastly, this indicates that MFA may get precipitated by the precipitation–adsorption method to reduce the amount of Cr in the wastewater.

### Effect of the adsorbent amount

**Fig. 7:**
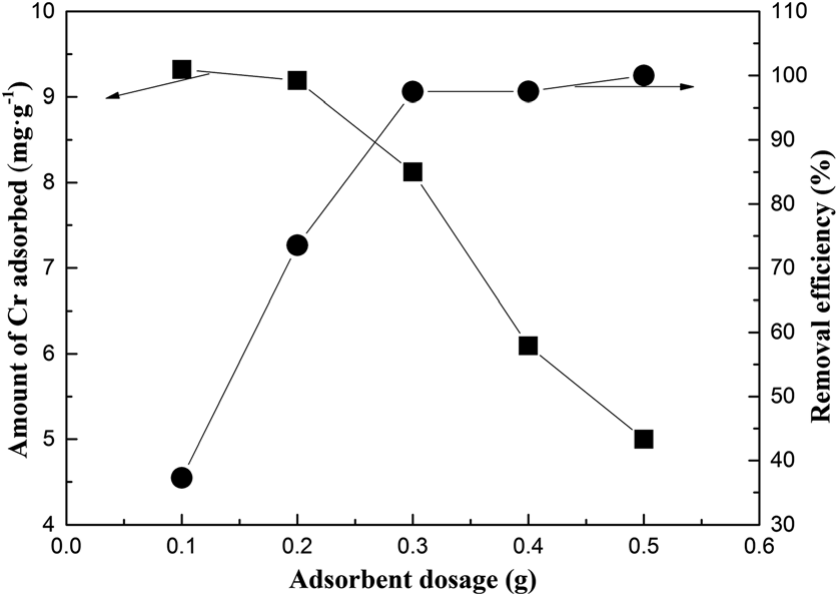
The effect of the dose of the adsorbent initially added on the adsorption of Cr on MFA (the initial concentration of Cr = 50 mg L^−1^; contact time = 2 h; and *T* = 15–25 °C).

Fig. 7 presents the effect of adsorbent dosage on the Cr-adsorption performance. Notably, the removal efficiency of Cr increased from 39.27% to 97.48% as the adsorbent dose increased from 0.1 to 0.3 g. Moreover, the removal efficiency of Cr increased rapidly since greater amount of adsorbent would imply greater surface areas for the adsorption of Cr.^31^ However, a further increase in the adsorbent mass (more than 0.3 g) resulted in an insignificant increase in the removal efficiency of Cr; this indicated that the excess amount of active sites was not fully utilized. Thus, the optimal FA dosage was estimated to be around 0.3 g.

### Effect of contact time

**Fig. 8:**
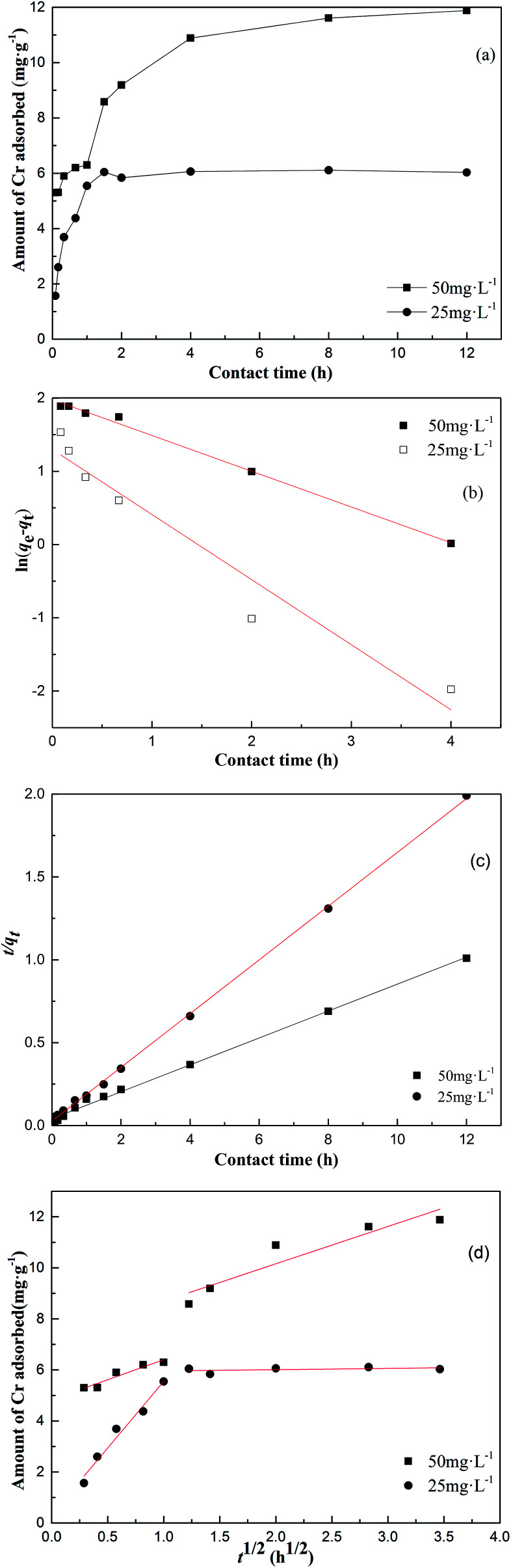
(a) The effect of contact time on the adsorption of Cr on MFA, (b) the linear fit obtained using the pseudo-first-order model, (c) the linear fit obtained using the pseudo-second-order model, and (d) the linear fit obtained using the intra-particle diffusion model.

The impact of contact time on the adsorption of 25 mg L^−1^ and 50 mg L^−1^ of Cr on the surface of MFA is presented in Fig. 8(a). The rate of metal removal was rapid in the first 120 min, and then, the rate gradually decreased until plateauing. In the initial stage, the adsorption was very swift due to the presence of a large number of active sites that were available for adsorption and a high concentration gradient of Cr between the adsorbent and the bulk solution. After sometime, the adsorption gradually decelerated because the number of vacant sites decreased and the Cr gradient also lessened. At the MFA dosage of 0.2 g, the adsorbent amount increased from 6.1 mg g^−1^ to 11.9 mg g^−1^ with an increase in the initial concentration of Cr from 25 mg L^−1^ to 50 mg L^−1^.

### Kinetic study

Fig. 8(b)–(d) display the linear plots of the kinetic models, and their corresponding data are compiled in Tables 1 and 2. The experimental data shows that the pseudo-second-order has higher correlation coefficients than the pseudo-first-order, and the value of the calculated equilibrium adsorption capacity *q*_e_ is close to the experimental *q*_e_ value. This proves that the process of adsorption of Cr on MFA follows the pseudo-second-order kinetic equation. Based on Fig. 8(d), there are two plots: the first and second plot represents film diffusion and intra-particle diffusion, respectively. Since *k*_id1_ > *k*_id2_, the rate of film diffusion was faster than that of the intra-particle diffusion. The plot of the intra-particle diffusion model did not pass through the origin; this revealed that the adsorption rate was not only affected by intra-particle diffusion, but also by film diffusion.

**Table 1:** The pseudo-first-order model and pseudo-second-order model kinetics fitting parameters for the adsorption of Cr onto MFA

*c*_0_ (mg L^−1^)	Pseudo-first-order model			Pseudo-second-order model		
	*q*_m_ (mg g^−1^)	*k*_1_ (h^−1^)	*R* ^2^	*q*_m_(mg g^−1^)	*k*_2_ (g mg^−1^ h^−1^)	*R* ^2^
25	3.66	0.8883	0.9374	6.1675	0.9835	0.9994
50	7.2112	0.4878	0.9951	12.3168	0.1595	0.9985

**Table 2:** The intra-particle diffusion model fitting parameters for the adsorption of Cr onto MFA

*c*_0_ (mg L^−1^)	Intra-particle diffusion model					
	*k*_id1_ (mg g^−1^ h^−0.5^)	*R* ^2^	*C*_1_ (mg g^−1^)	*k*_id2_ (mg g^−1^ h^−0.5^)	*R* ^2^	*C*_2_ (mg g^−1^)
25	5.2217	0.9634	0.3283	0.0498	0.938	5.9069
50	1.5739	0.8966	4.827	1.4630	0.8509	7.2315

**Fig. 9:**
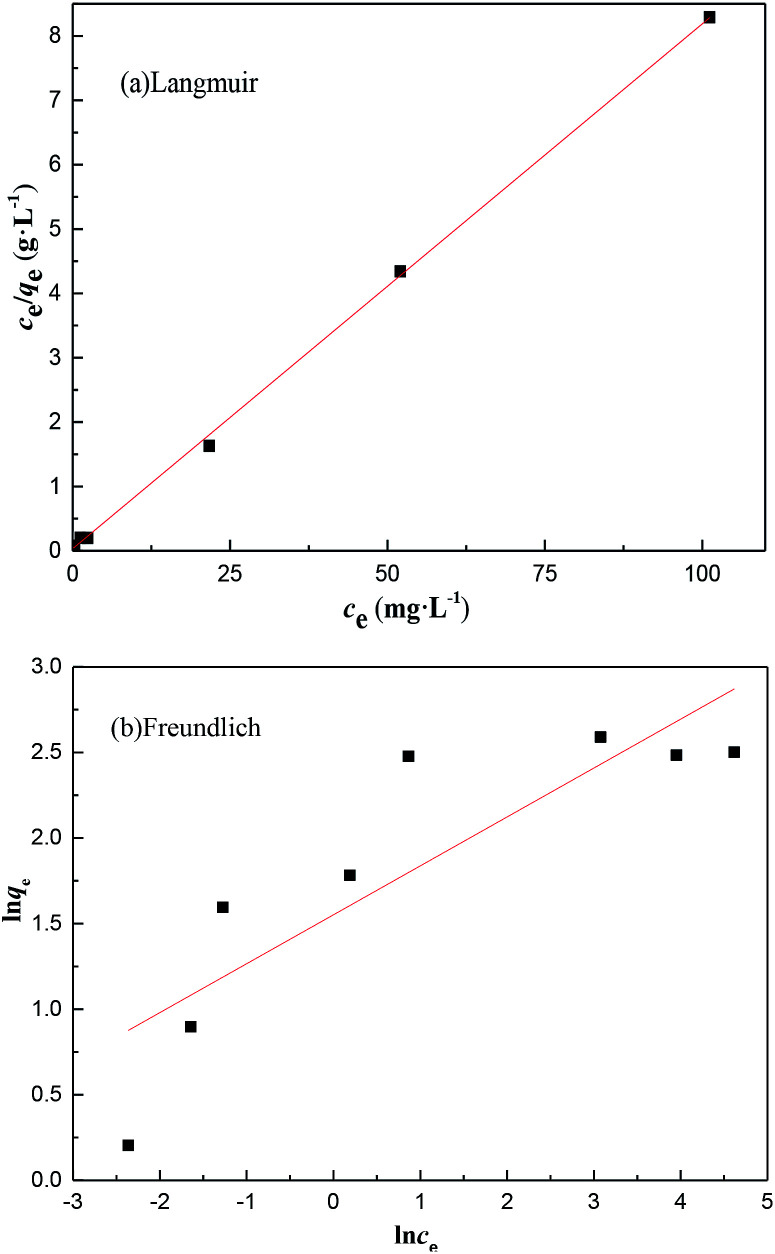
The (a) Langmuir and (b) Freundlich fitting curves for the adsorption of Cr on MFA.

### Isotherm study

The initial concentration of Cr provided the necessary driving force to overcome the mass transfer resistance, *i.e.* the higher the concentration of Cr in the wastewater, the greater the possibility of a clash between particles.^32^ The isotherm model parameters are presented in Table 3 and based on the value of the correlation coefficient (*R*^2^). The adsorption of Cr could be best described using the Langmuir isotherm, and the value *R*_L_ of the Langmuir equation was less than 1, which suggested that adsorption was favorable. Consequently, the adsorption of Cr on MFA can be represented appropriately by the Langmuir isotherm in the concentration range studied herein (Fig. 9).

**Table 3:** The Langmuir and Freundlich adsorption isotherm fitting parameters for the adsorption of Cr onto MFA

Langmuir isotherm		Freundlich isotherm	
*q*_m_ (mg g^−1^)	12.34	*K*_F_ (mg^(1−1/*n*)^ L^(1/*n*)^ ·g^−1^)	4.714
*K*_L_ (L mg^−1^)	2.45	1/*n*	0.285
*R* ^2^	0.9997	*R* ^2^	0.7122

## Conclusions

In this study, a two-step method of oxalic acid reduction-modified fly ash adsorption provided a promising low-cost and effective technology to remove Cr, which exhibited great potential for the treatment of Cr in wastewater.

Under sunlight, 1.5 g L^−1^ of oxalic acid acted as a reducing agent to reduce 100 mg L^−1^ of Cr(vi) to Cr(iii) in 7 days, thereby leaving a very stable Cr(iii) complex.

FA, the solid waste from coal-fired power plants, was utilized to adsorb Cr(iii). A combination of FA modified by 20 wt% of KOH, a contact time of 120 min, and a mass of 0.3 g of MFA led to a maximum removal efficiency (97.48%) of Cr(iii) at room temperature (15–20 °C).

Thus, the adsorption process followed the pseudo-second-order kinetic model, whereas the Langmuir isotherms provided better correlations for the isothermal adsorption process.

## Conflicts of interest

There are no conflicts to declare.

## Supplementary Material
